# Ethnic Variations in Central Corneal Thickness in a Rural Population in China: The Yunnan Minority Eye Studies

**DOI:** 10.1371/journal.pone.0135913

**Published:** 2015-08-14

**Authors:** Chen-Wei Pan, Jun Li, Hua Zhong, Wei Shen, Zhiqiang Niu, Yuansheng Yuan, Qin Chen

**Affiliations:** 1 Jiangsu Key Laboratory of Preventive and Translational Medicine for Geriatric Diseases, School of Public Health, Medical College of Soochow University, Suzhou, China; 2 The Second People’s Hospital of Yunnan Province, Kunming, China; 3 The First Affiliated Hospital of Kunming Medical University, Kunming, China; 4 First Affiliated Hospital of Nanjing Medical University, Nanjing, China; Zhongshan Ophthalmic Center, CHINA

## Abstract

**Purpose:**

To describe the ethnic differences in central corneal thickness (CCT) in population-based samples of ethnic Bai, Yi and Han people living in rural China.

**Methods:**

6504 adults (2119 ethnic Bai, 2202 ethnic Yi and 2183 ethnic Han) aged 50 years or older participated in the study. Each subject underwent standardized ocular examinations and interviewer-administered questionnaires for risk factor assessment. CCT was measured for both eyes using an ultrasound pachymeter. Regression and principal component analysis were performed to examine the relationship of ethnicity and other factors with CCT.

**Results:**

The mean CCT readings were 536.4 ± 34.2 μm in ethnic Bai, 532.1 ± 32.1 μm in ethnic Yi and 529.6 ± 32.7 μm in ethnic Han adults (P<0.001), respectively. There was a decreasing trend of mean CCT with increasing age across all ethnic groups. In multivariate linear regression models, increasing CCT was associated with younger age (*P*<0.001), male gender (*P*<0.001), Bai (*P*<0.001) or Yi (*P*<0.001) ethnicity, greater body mass index (*P*<0.001), higher systolic blood pressure (*P*<0.001), greater corneal curvature (*P*<0.001), deeper anterior chamber (*P* < 0.001), and thicker lens (*P*<0.001). Ethnicity contributed significantly to presence of thin cornea (60%; *P<* 0.001) compared with other factors. CCT had similar impact on intraocular pressure readings across all ethnic groups.

**Conclusions:**

This study of more than 6500 multiethnic participants demonstrates significant ethnic variations in CCT, with Han ethnicity having the thinnest cornea compared with ethnic minorities. These data are essential to guide future multiethnic clinical trials on CCT-related ocular conditions such as glaucoma.

## Introduction

Clinically, central corneal thickness (CCT) is an important ocular parameter, which could affect intraocular pressure (IOP) readings[1] and has been identified as an independent risk factor for glaucoma[2–6]. In addition, CCT has also been reported to be linked with many other clinical disorders such as retinal vein occlusion[7], optic disc hemorrhages[8], Parkinson's disease[9] and Turner syndrome[10]. Therefore, understanding the distribution and major determinants for CCT in general population is essential for both health care and clinical management.

Numerous population-based studies have reported the normative data of CCT among different ethnic groups and a potential ethnic variation has been suggested based on the results of these studies[11–16]. However, these results are difficult to interpret due to methodological disparities such as sampling strategies, age ranges of the study participants and machines used for CCT measurement among different studies. Thus, multiethnic studies using the same study protocols and modes for data collection across different ethnic groups are more valuable in understanding the inter-ethnic variations in CCT. Moreover, these data could provide further clues to the potential impact of genetic ancestry on CCT if all the ethnic groups reside in the same geographic location, which minimizes the effect of environmental exposures. However, these kind of multiethnic studies were few.

China is the world’s most populous country and the second largest economy with a multiethnic population including Han ethnicity and other 55 ethnic minorities. Han ethnicity is the major ethnic group which accounts for about 90% of the entire national population. The distribution and determinants of CCT have been well defined in Han ethnicity [13, 16, 17] but there were few studies addressing the issues of ethnic minorities. Thus, whether cornea is thicker or thinner in Han ethnicity compared with other ethnic minorities and whether there are any ethnic variations in the determinants of CCT remains uncertain. These data are essential to guide future multi-ethnic clinical trials on CCT-related ocular conditions such as glaucoma in China and may have implications for other countries with multiethnic populations.

Yunnan province, located at the southwest part of China, is a major residence for ethnic minorities in China. Besides the Han ethnicity, 25 ethnic minorities are reported to be residing in Yunnan province according to the census data 2010, which provides unique opportunities to investigate the ethnic variations in CCT in China. In this effort, we described the ethnic differences in CCT in population-based samples of ethnic Bai, Yi and Han people living in an inland rural community in Yunnan province.

## Materials and Methods

### Study cohort

The Yunnan Minority Eye Studies (YMES) are population-based studies conducted among different ethnic groups including the Han ethnicity (the major ethnic group) and other ethnic minorities in western China using the same study protocols. In a previous report, we have described the detailed methodology of YMES[18] and some major findings in a single ethnic group, that is, the Bai ethnicity [19–22]. Now, we have finished the data collection for the other two ethnic groups: Yi and Han ethnicities. Bai and Yi ethnicities are genetically heterogeneous. They have their own special cultures, inhabited environment, and languages. Most of them lived in Yunnan province located in southwest China and are mainly engaged in agriculture. Briefly, random cluster sampling strategies were adopted to select ethnic Bai, Yi and Han adults aged 50 years or older living in a rural community. Each village in the study site with a population of approximately 1000 was considered as a cluster during the sampling procedure. Villages with a population of less than 750 were combined and those of more than 1500 were divided and regrouped. Subsequently, 10% of the total clusters were randomly selected using a computer-assisted program. In the end of the study, 2133 (77.8%) ethnic Bai, 2208 (82.0%) ethnic Yi and 2205 (80.5%) ethnic Han adults participated in this study, respectively. Ethnicity was identified through official registry records. There were no age or gender differences between study participants and non-participants among all ethnic groups (all P > 0.05).

All studies were approved by the Kunming Medical University Institutional Review Board and the conduct of the studies adhered to the Declaration of Helsinki. Written informed consent was obtained from all participants.

### Clinical Examinations

CCT was measured for both eyes with an ultrasound pachymeter (Advent; Mentor O & O Inc, Norwell, Massachusetts) and the mean of the 5 readings was used for data analysis. Ocular biometric parameters including axial length (AL), anterior chamber depth (ACD), vitreous chamber depth (VCD) and lens thickness (LT) were measured using an Echoscan (US-800; Nidek Co., Ltd, Tokyo, Japan). Corneal curvature was measured using an autorefractor (RM-8000; Topcon Corp., Tokyo, Japan). Slit-lamp examination (model SL-1E; Topcon, Tokyo, Japan) was performed by trained study ophthalmologists to identify abnormalities of the anterior segment. Intraocular pressure (IOP) was measured using a handheld tonometer (TONO-PEN AVIA; Reichert Inc., New York, NY) device after instilling topical anesthesia (0.4% Benoxil; Oxybuprocaine, Santen, Japan).

Information regarding participants’ educational level, socioeconomic status, lifestyle-related factors (smoking, alcohol intake), disease history, and medication intake was collected using a detailed questionnaire by a trained research assistant. Height was measured in centimeters using a wall-mounted measuring tape after removing shoes while weight was measured in kilograms after taking off heavy clothing. Systolic blood pressure, diastolic blood pressure and pulse rate for all participants were recorded using a standardized mercuric-column sphygmomanometer, and one of four cuff sizes (pediatric, regular adult, large, or thigh) was selected based on the circumference of the participant’s arm. Diabetes mellitus was defined as non-fasting glucose levels higher than 200 mg/dL (11.1 mmol/L) or previous physician diagnosis of diabetes or use of anti-diabetic medications based on the American Diabetes Association guidelines[23].

### Statistical analyses

Data analyses were performed using STATA version 11.0 (StataCorp, College Station, Tex., USA). Study participants who had previous glaucoma-related laser or surgery, were using anti-glaucoma medication, underwent refractive surgery, or had corneal edema or dystrophy during the clinical examinations were excluded from analysis in this study. However, we included subjects with glaucoma history but without medication intake or surgical treatment in the current analysis.

One-way ANOVA test was performed to compare continuous variables across different ethnic groups for individual-level analysis. For eye-level analysis, repeated-measures ANOVA was performed. Logistic regressions with estimating equation were fit to compare categorical variables, accounting for the correlation between both eyes. Associations between factors of interest and CCT were assessed using univariate and multivariate generalized estimating equations. Besides age and sex, factors with a p value of less than 0.10 in the univariate model or factors of scientific importance were retained in the multivariate models. To determine whether ethnicity modified associations between factors of interest and CCT, a linear regression model was established with interaction terms between ethnicity and each potential risk factor, and a likelihood ratio test was performed on the interaction terms.

To elucidate how much a risk factor contributes to the ethnic variation in CCT, we performed a principal component analysis, which combined the major information from selected risk factors into a few independent components. The number of principal components extracted was determined based on cumulative proportion of the principal components which could explain at least 80% of total variability of the selected risk factors. A likelihood ratio test was performed to detect the residual effects of ethnicity which could not be explained from the selected factors, as indicated by the partial sum of square, which represents the relative variability explained by ethnicity or principal components within the model.

## Results

After excluding participants with previous history of glaucoma surgery or medication (n = 11), refractive surgery (n = 8) and corneal edema or dystrophy (n = 23), the data analysis included 6504 individuals (12802 eyes) including 2119 ethnic Bai, 2202 ethnic Yi and 2183 ethnic Han adults, who had CCT measurements in at least one eye. **Table 1**compares the demographic, systematic and ocular parameters among the three ethnic groups. In brief, adults of Bai ethnicity were youngest, best educated, heaviest, had the highest blood pressure; were least likely to smoke or take alcohol drinks, had the deepest anterior chambers, the thinnest lenses and the thickest corneas. Adults of Yi ethnicity were shortest, lightest, and were most likely take alcohol drinks. Adults of Han ethnicity had the lowest blood pressures, shortest ALs, narrowest anterior chambers and thinnest corneas.

**Table 1:** Demographic, systemic and ocular parameters among the three ethnic groups.

	Bai ethnicity (n = 2119)	Yi ethnicity (n = 2202)	Han ethnicity (n = 2183)	P value
**Demographic and systemic factors**				
Age (years)	64.4 (9.7)	65.0 (9.2)	65.4 (9.4)	0.004
Female gender	1352 (63.8)	1254 (56.9)	1307 (59.9)	<0.001
No formal education	759 (35.8)	1140 (51.8)	1174 (53.8)	<0.001
Body mass index (kg/m^2^)	21.9 (5.3)	20.5 (5.9)	21.7 (7.9)	<0.001
Height (cm)	158.2 (11.5)	155.3 (8.5)	156.2 (8.2)	<0.001
Weight (kg)	54.8 (9.9)	49.6 (14.3)	52.9 (20.1)	<0.001
Systolic blood pressure (mmHg)	145.6 (25.6)	143.0 (26.0)	140.0 (24.8)	<0.001
Diastolic blood pressure (mmHg)	88.2 (17.8)	87.5 (19.1)	86.0 (17.2)	<0.001
Diabetes	48 (2.3)	52 (2.4)	57 (2.6)	0.07
Smoking history	539 (25.4)	694 (31.5)	702 (32.2)	<0.001
Alcohol intake	316 (14.9)	591 (26.8)	451 (20.7)	<0.001
**Ocular biometric parameters**				
Corneal curvature (mm)	7.6 (0.01)	7.6 (0.01)	7.6 (0.01)	0.78
Axial length (mm)	23.1 (1.2)	23.0 (1.1)	22.9 (1.2)	<0.001
Anterior chamber depth (mm)	3.4 (0.5)	3.0 (0.4)	2.9 (0.4)	<0.001
Vitreous chamber depth (mm)	15.3 (1.4)	15.7 (1.2)	15.7 (1.4)	<0.001
Lens thickness (mm)	4.3 (0.6)	4.5 (0.4)	4.5 (0.5)	<0.001
Intraocular pressure (mmHg)	14.4 (3.3)	14.5 (2.4)	14.5 (3.1)	0.43
Central corneal thickness (μm)	536.4 (34.2)	532.1 (32.1)	529.6 (32.7)	<0.001

**Fig 1:**
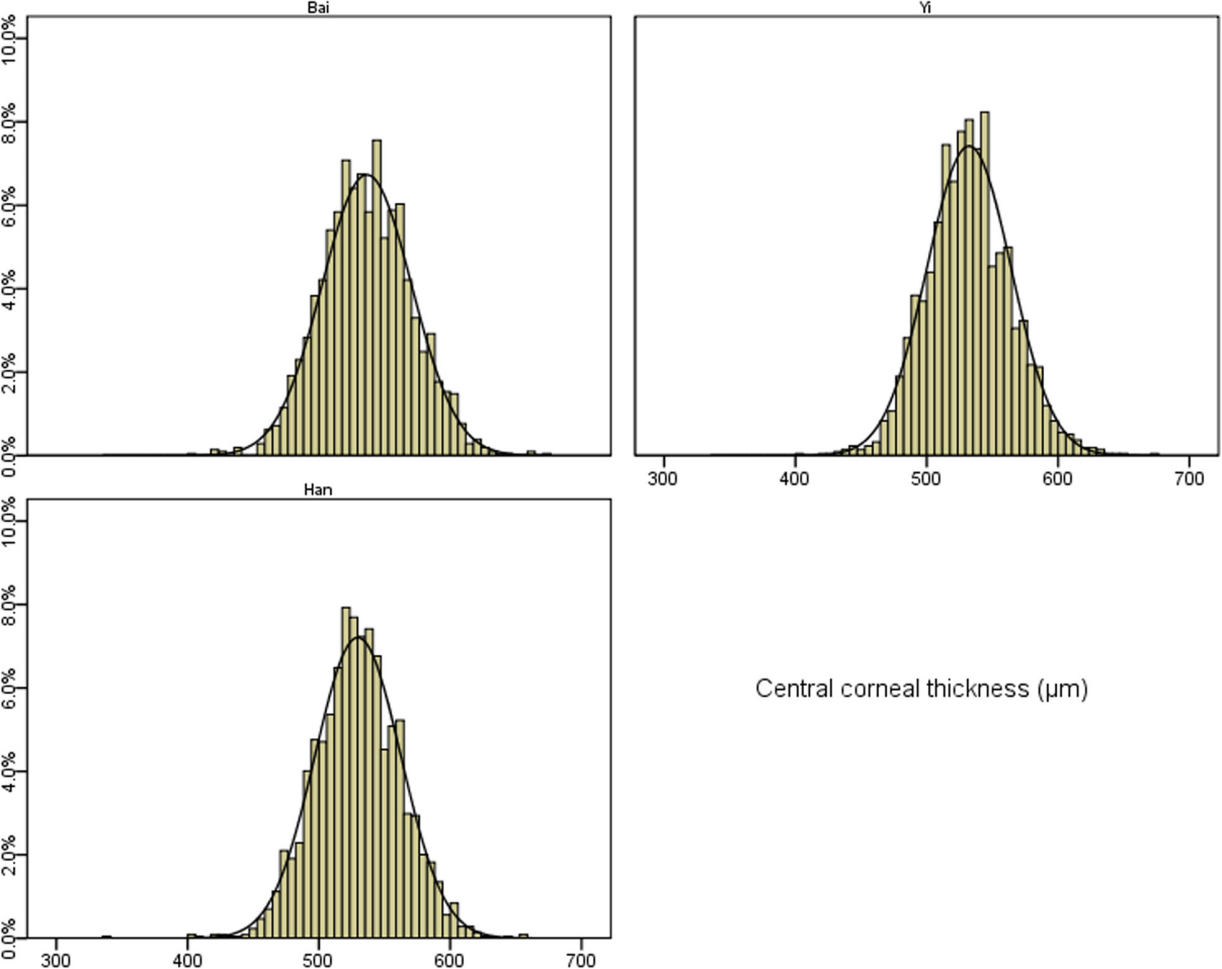
Distribution of central corneal thickness among the three ethnic groups.

**Fig 2:**
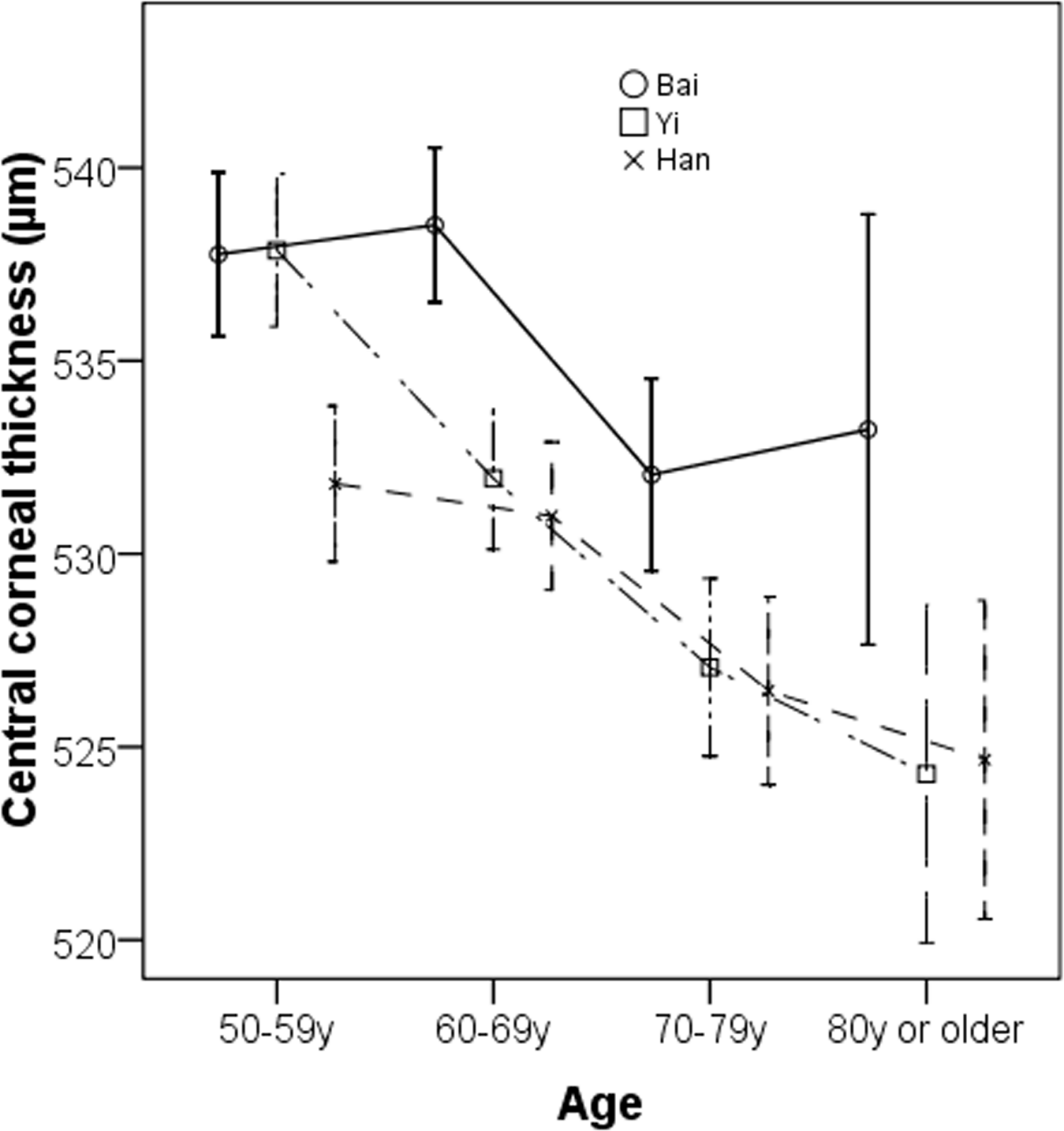
Age-specific mean central corneal thickness among the three ethnic groups.

**Fig 1**shows the distributions of CCT by different ethnic groups. Overall, the mean CCT readings were 536.4 ± 34.2 μm in ethnic Bai, 532.1 ± 32.1 μm in ethnic Yi and 529.6 ± 32.7 μm in ethnic Han adults (P<0.001), respectively. The skewness for the distribution of CCT was 0.07 in ethnic Bai, 0.14 in ethnic Yi and -0.07 in ethnic Han adults while the kurtosis was 0.21, 0.44, and 0.81 in these ethnic groups, respectively. Kolmogorov-Smirnov test revealed that CCT was normally distributed in all ethnic groups (all P > 0.05). Thin cornea defined as CCT of less than 555 μm was present in 70.2% (95% confidence interval [CI] 68.3%, 72.2%), 77.5% (95%CI 75.7%, 79.2%), 78.1% (95%CI 76.4%, 79.9%) eyes among ethnic Bai, Yi and Han adults, respectively. The age-specific mean CCT is shown in **Fig 2**. There was a decreasing trend of mean CCT with increasing age. The age-related pattern of CCT was more consistent in Yi and Han ethnic groups.

**Table 2**demonstrates the associations of demographic, systemic and ocular parameters with CCT. In univariate analysis, younger age (*P*<0.001), male gender (*P*<0.001), Bai (*P*<0.001) or Yi (*P*<0.001) ethnicity, greater body mass index (*P*<0.001), higher systolic blood pressure (*P*<0.001), greater corneal curvature (*P*<0.001), longer AL (*P*<0.001), deeper ACD (*P*<0.001), narrower VCD (*P*<0.001), and greater LT (*P*<0.001) were all significantly associated with an increasing CCT. Other factors such as education level, smoking history, alcohol intake, the presence of diabetes and lens status were not significant associates and therefore were not included in multivariate analysis (all *P* > 0.10). In multivariate linear regression models, increasing CCT was associated with younger age (*P*<0.001), male gender (*P*<0.001), Bai (*P*<0.001) or Yi (*P*<0.001) ethnicity, greater body mass index (*P*<0.001), higher systolic blood pressure (*P*<0.001), greater corneal curvature (*P*<0.001), deeper ACD (*P* < 0.001), and greater LT (*P*<0.001). No interaction effect was detected between ethnicity and other risk factors on CCT (all *P* > 0.05).

**Table 2:** Associations of central corneal thickness with systemic and ocular factors.

	Univariate analysis			Multivariate analysis		
	Regression coefficient	95% CI	P	Regression coefficient	95% CI	P
**Age (per 10 years)**	-3.35	-3.38, -3.32	<0.001	-3.52	-3.55, -3.49	<0.001
**Female vs. male**	-6.01	-6.03, -5.98	<0.001	-6.55	-6.60,-6.50	<0.001
**Ethnicity**						
Han	Reference			Reference		
Bai	6.78	6.72, 6.84	<0.001	6.56	6.49, 6.63	<0.001
Yi	2.49	2.43, 2.55	<0.001	2.11	2.05, 2.17	<0.001
No formal education	1.56	-0.45, 3.57	0.28		-	
Body mass index (kg/m^2^)	0.142	0.138, 0.145	<0.001	0.091	0.087, 0.095	<0.001
Systolic blood pressure (per 10 mmHg)	1.32	1.29, 1.35	<0.001	0.26	0.25, 0.27	<0.001
Diastolic blood pressure (per 10 mmHg)	0.02	-0.02, 0.08	0.22		-	
Diabetes (present vs. absent)	1.34	-2.68, 5.36	0.67		-	
Smoking history	3.02	-0.10, 6.14	0.11		-	
Alcohol intake	0.65	-2.29, 3.59	0.55		-	
Corneal curvature (mm)	4.83	3.23, 6.43	<0.001	2.95	2.06, 3.84	<0.001
Axial length (mm)	0.72	0.70, 0.74	<0.001	0.10	0,0.20	0.05
Anterior chamber depth (mm)	3.66	3.61, 3.70	<0.001	0.56	0.45,0.67	<0.001
Vitreous chamber depth (mm)	-0.27	-0.29,-0.25	<0.001	0.03	-0.06, 0.13	0.49
Lens thickness (mm)	1.50	1.45,1.55	<0.001	1.87	1.77, 1.98	<0.001
**Lens status**					-	
Phakia	Reference					
Pseudophakia	0.28	-1.35,1.91	0.31			
Aphakia	5.56	-2.86, 13.98	0.48			

CI = confidence interval

**Table 3**demonstrates the relative proportion of variance explained by ethnicity and principal components, which contains the information of other significant risk factors on mean CCT or thin cornea (CCT < 555μm). In the model of mean CCT, the principle component analysis achieved an 85.4% cumulative principal component proportion with 3 components extracted from the significant risk factors. After adjusting for these 3 components, the association of ethnicity with CCT remained significant (P < 0.001). Similarly, in the model of thin cornea (CCT < 555 μm), 87.2% cumulative principal component proportion was achieved with 4 components being extracted. Ethnicity was also significantly associated with the prevalence of thin cornea after adjusting for these 4 principal components (P<0.001).

**Table 3:** Principle component analysis one the association between ethnicity and other significant associated factors.

		Partial Sum of Square	Relative Proportion (%)	*P* Value	Cumulative Principle Component Proportion	Number of components extracted
**Mean CCT**						
	**Ethnicity**	52.32	56.5	<0.001	0.854	3
	**Principal components**	40.28	43.5	<0.001		
**CCT < 555** μm						
	**Ethnicity**	59.45	60.0	<0.001	0.872	4
	**Principal components**	39.56	40.0	<0.001		

IOP readings were significantly affected by CCT among all ethnic groups, even if cornea curvature was adjusted for. Each 10 μm increase in CCT is associated with 0.12 mmHg (95%CI: 0.11, 0.14) increase in IOP readings in ethnic Bai adults, 0.13 mmHg (95%CI: 0.11, 0.14) increase in IOP readings in ethnic Yi adults and 0.12 mmHg (95%CI: 0.11, 0.13) increase in IOP readings in ethnic Han adults, respectively. The results were similar if glaucoma patients were removed from analysis.

## Discussion

This multiethnic study among adults aged 50 years or older of three various ethnicities residing in the same geographical location provided novel data for precise comparison of ethnic disparities in CCT, a key ocular predictor for glaucoma, in China. Adjusting for other systemic and ocular factors, adults of Han ethnicity had the thinner corneas compared with ethnic minorities such as adults of Bai or Yi ethnicities. Although ethnicity was significantly related to CCT, the selected systemic and ocular factors in this study cannot fully explain the entire ethnic variation in CCT, suggesting other factors such as genetic factors, which were not captured in this study, may also be important to explain the ethnic variations in CCT.

The most important finding for our study is that people of different ethnicity have different thickness of corneas, even if they have been exposed to the same environment. This finding is consistent with the result of a previous report in Singapore, a city state located on the equator, which showed Chinese had the thickest corneas as compared with Malays or Indians[14]. However, many of the study participants in the Singapore study were first generation immigrants from China, Malaysia and India, who may have different environmental exposures at their early life[24]. Therefore, whether environmental exposures at early life could have a major impact on CCT remains unclear. Our study subjects were not migrants, almost all of whom were born in the community where the study site was located. Therefore, our results may be powerful in understanding the effect of genetic ancestry on CCT. In addition, we performed a principal component analysis in order to better understand the relative contribution of ethnicity to the variations in CCT while considering the effect of inter-ethnic differences in many other factors including both factors included and not captured by our study. Although the principal component analysis confirmed that ethnicity was significantly related to CCT, the proportion of the effects of ethnicity on CCT unexplained by the selected risk factors in the analysis was considerable (more than 40%; **Table 3**). The interpretation of these results is that other risk factors, which were not captured by this study (e.g., genetic factors), may also have a great impact on CCT.

Population-based studies have reported a wide range of estimates for CCT across different ethnic groups, especially in Asians, in different geographic locations. For example, mean CCT was reported to be 530.9 μm in Koreans[25], 521.0 μm in Japanese[12], 535.6 μm in Chinese living in northern China[17], 541.5 μm in Chinese living in southern China[13], 514.0 μm in Indians living in India[15], 540.9 μm in Singapore Malays[14], 552.3 μm in Singapore Chinese[14] and 540.4 μm in Singapore Indians[14]. These figures were difficult to interpret due to the disparities in study protocols including sampling strategies, methods for CCT measurements (ultrasound pachymetry vs. specular microscopy), age ranges of the study participants and so on. Therefore, direct comparison between our study and studies conducted by other research groups may be invalid.

With regard to the determinants of CCT, most of the determinants identified in this study have been reported and discussed previously[12, 14, 15]. We indicated that the major determinants for CCT are systemic factors such as blood pressure and body mass index or ocular biometric parameters such as ACD, LT and cornea curvature. Socioeconomic factors (e.g. educational level) or lifestyle-related factors (e.g. smoking, alcohol intake) had little impact on CCT. These findings implied that genetic ancestry may be more important for CCT as compared with environmental exposures. In addition, we did not detect any interaction effect between ethnicity and any of the determinants for CCT, indicating that the impact of these factors on CCT may be equally important to each ethnic group.

Some clinical implications from our study should be noted. The Ocular Hypertension Treatment Study has revealed that untreated patients with a CCT of less than 555 μm had a 3-fold higher risk for developing glaucoma as compared with their counterparts who had a CCT of more than 588 mm[26]. Our studies demonstrated that the prevalence of thin cornea (defined as CCT less than 555μm) is extremely high in rural China (70% in ethnic Bai adults, 78% in ethnic Yi or Han adults). Therefore, as recommended by the Ocular Hypertension Treatment Study, treatment for ocular hypertension should be reserved only for patients with thin corneas, who may be at a higher risk of developing glaucoma[26]. In addition, data from our study provided the normative data for CCT in ethnic minorities in China and are important to guide future multi-ethnic clinical trials on CCT-related ocular conditions such as glaucoma. The findings from this study may also be related to many other countries with multiethnic populations and a heavy burden of glaucoma for health resource allocation and clinical management among different ethnic groups.

The study’s strengths included a large sample size, multiethnic study participants, reasonable response rates, standardized clinical measurements for CCT and the usage of the same study protocols for data collection across different ethnic groups. There were also some limitations for this study, which should be acknowledged. As indicated by the principal component analysis, we were unable to identify the full set of explanatory factors for the observed ethnic differences in CCT. In addition, ethnicity was identified by the official records in this study. Genetic measures of ancestry may be more accurate for ethnicity identification but was not performed in this study due to limited resources. Finally, the cross-sectional design limited the ability to assess causal relationship when analyzing risk factors.

In conclusion, this study of more than 6500 multiethnic participants living in the same geographic location in rural China demonstrates significant ethnic variations in CCT, with Han ethnicity having the thinnest cornea as compared with ethnic minorities. Ophthalmologist should be aware of the ethnic variations in CCT across ethnic groups and the treatment strategies for glaucoma should be targeted for each ethnic group individually rather than in aggregate. Investigators should also take the ethnic variations into consideration when designing multi-ethnic clinical trials on CCT-related ocular conditions.
